# Supplementary material for: Parents’ views on accepting, declining, and expanding newborn bloodspot screening
Source: PLoS One. 2022 Aug 18;17(8):e0272585. doi: 10.1371/journal.pone.0272585 (PMC9387838; doi:10.1371/journal.pone.0272585)
Supplement: S1 Appendix — (DOCX) [file pone.0272585.s001.docx]

**S1 Appendix. Questionnaire used in this study.**

**Welcome to the questionnaire about the heel prick test**

**Do you want to participate?**

You have received a letter about the heel prick test questionnaire.

The letter explains why we made the questionnaire and what happens with the answers and your personal details.

Please tick one of the following boxes to indicate whether you want to participate or not

0 Yes, I have read the letter and I am participating

0 No, I am not participating in this questionnaire

If you want, you can indicate why you are not participating:------------------------------------------------------------------------------------------------------------------------------------------------------------------------------------------------------------------------------------------------------------------------------------------------------------------------------------------------------------------------------------------------------------------------------

1. **Questionnaire on the heel prick test**

This is a questionnaire about the heel prick test. When a baby is a few days old, he or she will be offered a heel prick test. With the heel prick test a few drops of blood are taken from the baby's heel.

With the heel prick test, we find out if a baby has a rare but serious health condition. Early treatment can improve the baby’s health, and prevent severe disability or even death. The Dutch government pays for and arranges the heel prick.

We would like to hear your opinion about the heel prick test.

Thank you for participating!


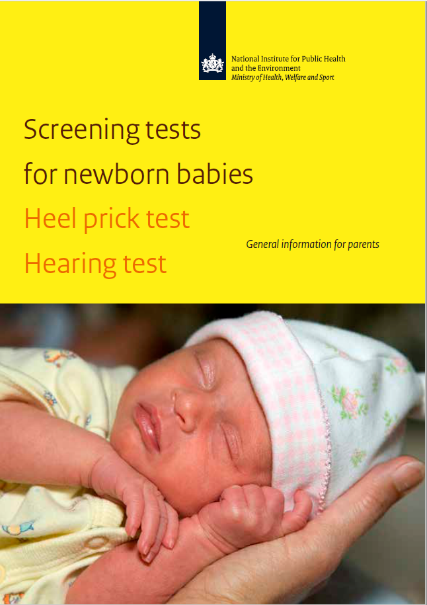


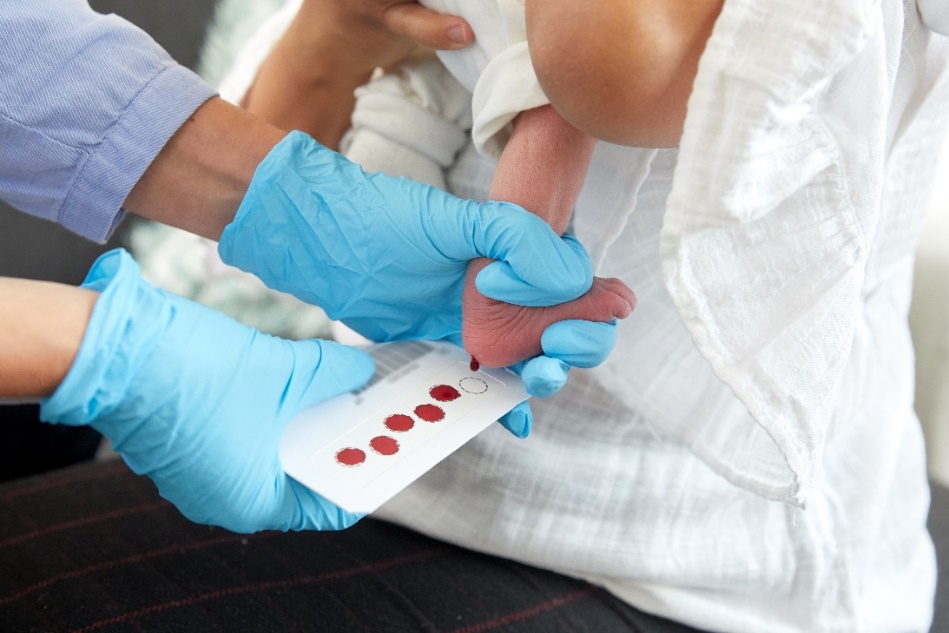


Who is completing this questionnaire? Please tick the correct box:

- Mother
- Father
- Mother and Father
- Someone else, namely (specify):………………………………………

Please tick:

1. Was the heel prick test carried out on your baby?

- Yes
- No ***[go to question 3]***
- Other, namely (specify): ………………………….***[go to question 3]***

1. Where was the heel prick test carried out?

- At home
- In the hospital
- Somewhere else, for example abroad, or a birth center (specify) ……………………………………………………………………………………

1. What was the most important reason for you to participate / not participate in the heel prick test?………………………………………………………………………………………

……………………………………………………………………………………………………………………………………………………

1. You did or did not participate in the heel prick test. Indicate how important the following reasons were for you in making a decision about participation in the heel prick test.

|  | Not at all important | Not important | Neutral | Important | Very important |
| --- | --- | --- | --- | --- | --- |
| a. Because the heel prick test reassures me |  |  |  |  |  |
| b. Because I can prevent my child from getting health complaints from a disorder |  |  |  |  |  |
| c. Because I think the heel prick test is painful for my child |  |  |  |  |  |
| d. Because of my faith or religion |  |  |  |  |  |
| e. Because of my view of life (for example anthroposophical or naturopathic) |  |  |  |  |  |
| f. How my data and my child’s data are handled |  |  |  |  |  |
| g. Because the government arranges and pays for the heel prick test |  |  |  |  |  |
| h. Because I am confident that the results of the heel prick test are reliable |  |  |  |  |  |
| i. Because of Corona-virus |  |  |  |  |  |

1. Below you see a number of statements about the heel prick test. Please state after each statement whether you think it is "true" or "not true". It is important that you answer what you think. You don't have to look up information or ask others.

|  | True | Not true | Don’t know |
| --- | --- | --- | --- |
| a. The conditions detected by the heel prick test have serious consequences if they are not treated. |  |  |  |
| b. The chance that a child has one of the ‘heel prick test conditions’, is very small. |  |  |  |
| c. If the results of the heel prick test are abnormal, it is not yet certain that a child has the condition. More investigations are needed before this is certain. |  |  |  |
| d. A normal result of the heel prick test is a guarantee that the child is completely healthy. |  |  |  |
| e. The heel prick test is a reliable test because children with one of those conditions are almost always diagnosed. |  |  |  |
| f. If the results of the heel prick test are uncertain, an extra blood sample is needed. |  |  |  |

1. What is your opinion about the heel prick test? Indicate your opinion by answering the following five questions (a - e) and ticking one of the boxes.

***For example:***

*If you find maternity care important, tick the box that is closest to ‘important’. If you feel that maternity care is less important, you tick a box more to the left, nearer ‘not important’. Please apply this to each word pair and tick one box only for each pair.*

|  | I find maternity care…. | | | | | | |
| --- | --- | --- | --- | --- | --- | --- | --- |
| a. | Not important | □ | □ | □ | *X* | □ | Important |
| b. | Useless | □ | □ | *X* | □ | □ | Useful |

| I find the heel prick test for my baby: | | | | | | | |
| --- | --- | --- | --- | --- | --- | --- | --- |
| a. | Bad |  |  |  |  |  | Good |
| b. | Useless |  |  |  |  |  | Useful |
| c. | Scary |  |  |  |  |  | Not scary |
| d. | Not reassuring |  |  |  |  |  | Reassuring |
| e. | Annoying |  |  |  |  |  | Pleasant |

1. Indicate to what extent you agree with the following statements about the heel prick test:

|  | Completely disagree | Disagree | Neutral | Agree | Completely agree |
| --- | --- | --- | --- | --- | --- |
| a. The heel prick test is just part of life. |  |  |  |  |  |
| b. I was in doubt about participating in the heel prick test. |  |  |  |  |  |
| c. I received sufficient information about the heel prick test so that I could make a well-founded decision about whether or not to participate. |  |  |  |  |  |
| d. I gave it a long time whether I should participate in the heel prick test or not. |  |  |  |  |  |
| e. I trust that the government will inform me adequately about the heel prick test. |  |  |  |  |  |

**B. New conditions in the heel prick test**

1. The heel prick test is used to test for several conditions. In the future, the heel prick will test for even **more** conditions. Do you think that is a good idea? Please tick the correct box and fill in the reason why.

- Yes, because…………………………………………………………………………………

………………………………………………………………………………………………………

………………………………………………………………………………………………………

- No, because………………………………………………………………………………..

………………………………………………………………………………………………………

………………………………………………………………………………………………………

- I don’t know, because....................................................................................................

………………………………………………………………………………………………………

………………………………………………………………………………………………………

1. Do you think it is a good idea if the following conditions are added to the heel prick test?

|  | Very bad idea | Bad idea | Neutral | Good idea | Very good idea |
| --- | --- | --- | --- | --- | --- |
| a. A condition of which it is uncertain whether your child will get any complaints. |  |  |  |  |  |
| b. A condition which treatment can cause serious side effects for your child. |  |  |  |  |  |
| c. A condition for which some of your child’s DNA (the genetic material) needs to be tested. |  |  |  |  |  |
| d. A condition for which there is no treatment or no medication. |  |  |  |  |  |
| e. A condition which will give your child symptoms only later in life. |  |  |  |  |  |
| f. A condition in which the result could indicate that the child is not sick but that the mother is sick. |  |  |  |  |  |
| g. A condition with which your child can lead a normal life, such as going to school, participate in sports etc. even without treatment. |  |  |  |  |  |

**[ “adrenoleukodystrophy (ALD) scenario”** ]

1. Sometimes the symptoms of a condition are different in boys than in girls.

For example:

In boys:

- the condition causes problems in the brain and adrenal glands that may cause death if treatment is not started on time.

- the condition can be adequately treated if discovered early.

In girls:

- the condition causes different and less major problems later in life (between 40-60 years of age) such as difficulties in walking.

- The condition cannot be treated..

Do you think it is a good idea to test for this condition with the heel prick test? (tick only 1 answer)

- Yes, I think it is a GOOD idea to test for these conditions, but ONLY in BOYS because (specify): ………………………………………………………………………………………………………………………………………………………………………………………………
- Yes, I think it is a GOOD idea to test for these conditions in BOTH BOYS and GIRLS because (specify): ………………………………………………………………………………………………………………………………………………………………………………………………
- No, I do NOT think it is a good idea to test for these conditions because (specify):

……………………………………………………………………………………………………………………………………………………………………………………………...

4. Do you have any further remarks about the new conditions in the heel prick test (questions 1 to 3)?

**………………………………………………………………………………………………………………………………………………………………………………………………………………………………………………………………………………………………………………………………………**

**C. A few final questions about you**

The following questions are about yourself. In case you fill in this questionnaire together with your partner, we ask you to enter *the mother’s* details below.

|  | My age is ….. years | |
| --- | --- | --- |
|  |  | |
|  | I am:   - married - cohabitant - single - Other,(specify) :………………………………………………………….. | |
|  |  | |
|  | My education:   - Elementary school - Lower level of secondary school - Lower vocational training - Higher level of secondary school - Intermediate vocational training - Higher Vocational Training - University - I don’t know - Other, (specify): | |
|  | Are you active within your religion? | |
|  | - I am not religious - I am not active within my religion - I am a scarcely active within my religion - I am active within my religion |  |
|  |  |  |
|  | Are the following subjects important to you? You can tick more than one box. | |
|  | - Anthroposophy - Homeopathy - Natural medicine - None - Other (specify)…………………………………………………………… |  |
|  |  | |
|  | The language I speak at home is:   - Only Dutch - Dutch and another language - Another language…………………………………………………. | |
|  |  | |
|  | The first 2 numbers of your postal code are (you can skip this question if you want)  _ _ x x | |
|  |  | |
|  | The mother of the new born baby was born in:   - The Netherlands - Turkey - Morocco - Surinam - Dutch Antilles/Aruba - Other, (specify) …………………………………………… | |
|  |  | |
|  | The father of the new born baby was born in:   - The Netherlands - Turkey - Morocco - Surinam - Dutch Antilles/Aruba - Other, (specify) | |
|  |  | |
|  | The grandparents of the new born baby were born in:   - None of the grandparents were born in the Netherlands - 1 was born in the Netherlands - 2 were born the Netherlands - 3 were born in the Netherlands - All were born in the Netherlands - Other, (specify)…………………………………………….. | |
|  |  | |
|  | Do you have any other children?   - No - Yes, number of children (specify):…….. | |

The following questions are about your new born baby:

|  | How old is your child at the moment (or your children, if they are twins)?  You can enter the age in weeks or in months.  …….. weeks (specify)  ………months (specify |
| --- | --- |
|  |  |
|  | What is the sex of your baby?   - Boy - Girl - Twins ( twins, triplets etc): …… boys ….... girls (specify numbers) - Other (specify)…………………………………………….. |
|  |  |
|  | After how many weeks pregnancy was your baby (or were your twins) born?   - Less than 37 weeks - 37 weeks or more - I don’t know |
|  |  |
|  | What was the birth weight of your baby (or twins)?   - 2500 gram or less - More than 2500 gram - I don’t know |
|  |  |
|  | Are you planning to give your baby vaccinations (for example diphtheria, whooping cough, tetanus and polio)?   - Yes, all vaccinations - Yes, but not all the vaccinations - No - Other specify)…………………………………………………………………. |

17. Do you have any final remarks about the questionnaire? Please write them down below:
