# Supplementary material for: Parents’ views on accepting, declining, and expanding newborn bloodspot screening
Source: PLoS One. 2022 Aug 18;17(8):e0272585. doi: 10.1371/journal.pone.0272585 (PMC9387838; doi:10.1371/journal.pone.0272585)
Supplement: S1 Table — (DOCX) [file pone.0272585.s002.docx]

**S2 Table. Number of respondents (who participated in NBS and those who declined NBS) who correctly answered the knowledge questions.^a^**

|  | Respondents who participated in NBS  n = 804 | Respondents who declined NBS  n = 48 |
| --- | --- | --- |
| A normal result of the heel prick test is a guarantee that the child is completely healthy. (false), n (%) | 751 (93) | 39 (81) |
| The conditions detected by the heel prick test have serious consequences if they are not treated. (true), n (%) | 672 (84) | 27 (56) |
| If the results of the heel prick test are abnormal, it is not yet certain that a child has the condition. More investigations are needed before this is certain. (true), n (%) | 653 (81) | 38 (79) |
| The chance that a child has one of the ‘heel prick test conditions’, is very small. (true), n (%) | 626 (78) | 40 (83) |
| The heel prick test is a reliable test because children with one of those conditions are almost always diagnosed. (true), n (%) | 620 (77) | 14 (29) |
| If the results of the heel prick test are uncertain, an extra blood sample is needed. (true), n (%) | 401 (50) | 11 (23) |
| Knowledge score^b^, mean (SD) | 7.72 (1.86) | 5.87 (2.60)* |

^a^ Arranged from question most correctly answered to question least correctly answered (maximum of 1 missing value).

^b^ Knowledge score: all 6 knowledge items recoded into a score ranging from 0 (none correct) to 10 (all correct).

* P<.001 (non-parametric Mann-Whitney U test).
