# Supplementary material for: Parents’ views on accepting, declining, and expanding newborn bloodspot screening
Source: PLoS One. 2022 Aug 18;17(8):e0272585. doi: 10.1371/journal.pone.0272585 (PMC9387838; doi:10.1371/journal.pone.0272585)
Supplement: S2 Table — (DOCX) [file pone.0272585.s003.docx]

**S3 Table. Attitude towards NBS screening by participation.**

| I find the heel prick test for my child: | Respondents who participated in NBS^a^  Mean (SD) | Respondents who declined NBS^b^  Mean (SD) |
| --- | --- | --- |
| 1= bad - 5 = good | 4.76 (0.58) | 2.62 (1.15) |
| 1 = useless - 5 = useful | 4.49 (1.15) | 2.55 (1.08) |
| 1 = not reassuring - 5 = reassuring | 4.26 (0.85) | 2.20 (1.14) |
| 1= scary - 5 = not scary | 3.99 (1.14) | 3.49 (1.33) |
| 1 = annoying - 5 = pleasant | 3.32 (1.05) | 1.89 (0.99) |
| Attitude-score^c^ | 8.33 (1.25) | 5.16 (1.64) * |

^a^The n slightly differed between items (n = 800 to 802).

^b^The n slightly differed between items (n = 45 to 47).

^c.^ Attitude score: all 5 attitude items recoded into a score ranging from 2 (all negative attitudes) to 10 (all positive attitudes).

* P<.001 (non-parametric Mann-Whitney U test).
