# Supplementary material for: Parents’ views on accepting, declining, and expanding newborn bloodspot screening
Source: PLoS One. 2022 Aug 18;17(8):e0272585. doi: 10.1371/journal.pone.0272585 (PMC9387838; doi:10.1371/journal.pone.0272585)
Supplement: S3 Table — (DOCX) [file pone.0272585.s004.docx]

**S4 Table. Undisputed choice to participate in NBS or decline NBS.**

|  | Respondents who participated in NBS  Mean (SD)  n = 804 | Respondents who declined NBS  Mean (SD)  n = 48 |
| --- | --- | --- |
| The heel prick test is just part of life | 4.22 (0.83) | 1.69 (0.99) * |
| I gave it a long thought whether I should participate in the heel prick test or not | 1.26 (0.61) | 2.90 (1.33) * |
| I was in doubt about participating in the heel prick test | 1.24 (0.60) | 3.58 (1.38) * |

1 = completely disagree – 5 = completely agree.

* P-value < .001 (Chi square test for ordinal trend).
