# Supplementary material for: Parents’ views on accepting, declining, and expanding newborn bloodspot screening
Source: PLoS One. 2022 Aug 18;17(8):e0272585. doi: 10.1371/journal.pone.0272585 (PMC9387838; doi:10.1371/journal.pone.0272585)
Supplement: S4 Table — (DOCX) [file pone.0272585.s005.docx]

**S5 Table. NBS knowledge and attitude of low-/middle- versus high-educated parents that participated in NBS.**

|  | Low-middle educated^a^  Mean (SD)  n = 202^b^ | High educated^a^  Mean (SD)  n = 602^b^ | Difference  (p-value)^c^ |
| --- | --- | --- | --- |
| Knowledge-score (0 to 10 (all correct)) | 7.15 (1.98) | 7.91 (1.78) | -0.75 (<.001) |
| Attitude-score (2 to 10 (all very positive)) | 7.98 (1.44) | 8.44 (1.16) | -0.46 (<.001) |

^a^ Low education level = Elementary school, lower level of secondary school, and lower vocational training. Middle education level = Higher level of secondary school and intermediate vocational training. High education level = High vocational training and university.

^b^Attitude-score had 1 missing value in the low-middle educated group, leaving n = 202, and 2 missing values in the high educated group, leaving n = 602.

^c^Non-parametric Mann-Whitney U test.
