# Supplementary material for: Parents’ views on accepting, declining, and expanding newborn bloodspot screening
Source: PLoS One. 2022 Aug 18;17(8):e0272585. doi: 10.1371/journal.pone.0272585 (PMC9387838; doi:10.1371/journal.pone.0272585)
Supplement: Attachment — Submitted filename: Peer review for PLOS ONE.docx [file pone.0272585.s006.docx]

Review of manuscript submission to PLOS ONE

Parents’ views about newborn bloodspot screening (non-)participation and expansion

**Overall**

This is an excellent article overall and I particularly appreciate the efforts to recruit and gain insight into reasons for non-participation in NBS!

The introduction and discussion would be enhanced by inclusion of more recent articles in the literature; for example, those which have explored perspectives of parents with children who were diagnosed at NBS for conditions with late-onset subtypes and the ethical quandaries in detection of these at birth (see suggestions below).

Also, analytical statistics need to be performed for study results involving comparisons between groups, in order for them to be accepted as not obtained due to chance (significant). I know that your sample size is low for the non-participating group, but such analysis can be done using certain statistical techniques and vastly increases the conclusions you can draw from your data. Otherwise, there are certain statements / conclusions you just can’t scientifically make.

**Abstract**

Please reword the 1^st^ sentence is the results subsection of the abstract to make clear that these are the most important reasons reported by the parents in the study.

You cannot make the comparisons between groups in the 2^nd^ and 3^rd^ sentences of the abstract results without having analytical statistics to back up your claim.

In the final section of the abstract results, please reword to say that “Most parents in the NBS-participating group agreed……”

In the abstract conclusions section, please reword to say that “Most parents in the NBS-participating group made an informed decision……” Might include a sentence about the non-participating group’s informed decisions not to participate as well, since this is new data and relevant to your results.

**Introduction**

The van Dijk article citation is excellent, but a lot of the other literature cited on parental perspectives on NBS in the Introduction is dated. Recent articles have been published exploring the perspective of parents whose children’s conditions were actually detected via NBS (e.g. Pruniski et al 2018’s Newborn screening for Pompe disease: Impact on families)(White et al 2021’s Absorbing it all – a meta-ethnography of parents’ unfolding experiences of NBS)(Kariyawasam et al 2021’s We needed this)(etc.). In addition, a few recent articles have asked adults with genetic conditions whether they would have wanted to know their condition at birth via NBS (e.g. Lisi et al’s 2016 & 2021 articles). Please update your literature review to include some of these.

**Methods**

Page 5, lines 95-99 - Are the #’s of parents who declined participating in NBS in 2019 and 2020 from the entire year or just the five-weeks-earlier period the parents who consented to NBS were taken from? It’s not clear from the wording in the text – please clarify.

Page 6, line 111 – Please clarify whether the 5 parents the questionnaire was tested on had allowed their children to have NBS or declined it, whether their children had positive NBS results or whether all had received negative NBS results, whether or not the 5 parents also participated in the study, etc.

Page 7, lines 148-151 – Tables S1, S2, and S3 allow the reader to see the questions asked of the participant. It would be nice to see the Views on current NBS questions as well. Consider adding a copy of the entire survey in an Appendix for reader reference.

Page 8, line 153 – Please include which 7 conditions participants were asked to evaluate for expanded NBS, if these questions were about specific genetic conditions. If they are about more general scenarios (as Table 5 suggests), please make that clear. In general, the questions asked seem to be spread out over a large # of tables. Again, consider consolidating them all by placing a copy of the entire survey in an Appendix for ease of reader reference.

**Results**

Please clarify the #s in the “Statistics” column in Table 1. As stated now (%s), I do not know how to interpret them. Are they simply meant to reflect the % of each row out of the total possible? If so, why do we not see them for every row? If they are meant to represent something else, what else? A p-value to determine whether there were any pre-existing significant differences between groups would lend greater credibility to your results.

Also in Table 1, please define how you categorized someone as having “low/middle” levels of education versus “high” education.

Table 2: Please include p-value or similar analytical statistic to allow reader to determine whether difference between columns is significant or not.

Page 13, lines 236-237 – You cannot conclude that respondents who participated in NBS made a more undisputed choice to participate or that NBS non-participants indicated more doubt without some form of analytical analysis to show that the difference in score was not due to chance. Please do analysis, as was done in Table S5 (also, please define the terms “low-middle educated” and “high-educated in Table S5).

**Discussion**

Page 18, lines 287-289 - The beginning of the discussion states that overall respondents were very positive about the current NBS program, as well as expanded screening. However, this is confounded by the vastly different sample sizes between those who opted in for NBS vs those that opted out. You can reword this to say that those participants who opted into NBS were very positive about the current NBS system, while those who opted out felt differently (but you have to do analytical statistics to show the differences between groups were not obtained by chance).

Page 18, lines 289-291 – Likewise, in order to make the statement here regarding the proportion of those making an informed decision being lower in one group than the other, a p-value or other analytical statistic is needed to prove that the results were not obtained by chance. The only part of this statement that has been statistically analyzed is the education part (Table S5).

Page 19, line 294 – Would recommend rewording this sentence to “The reason respondents reported as most important to participate in NBS was…..,” so as to make clear it is the respondents’ opinion, not the authors. This is well done at the beginning of the 2^nd^ paragraph on this page, so maybe copy that wording for the 1^st^ paragraph as well.

Page 19, lines 303-304 – Cannot say that “Non-participating parents were more actively religious…..compared to participating parents” without a p-value to prove this.

Page 19, lines 312-314 - Cannot say that “Non-participating parents were more often in doubt….compared to NBS participants” without a p-value to prove this.

Page 20, line 339 – It looks as though you have only included the group that made the informed decision to participate in this % (83%). Wouldn’t it be more accurate to include those who made the informed decision NOT to participate in this # as well? Either that or change the wording of the sentence to make clear that you are only referring to the NBS-participating group, not the entire group of participants.

Page 21, line 344 – You need a p-value to be able to scientifically declare this a difference between groups.

Page 21, line 355 - You need a p-value to be able to scientifically declare this a difference between groups.

Page 21-23, section on “Parents’ views on expanding Dutch NBS” – This section does a much better job of incorporating some newer article citations – love it A few others which are relevant and add a different perspective include O’Connor et al’s 2018 Psychosocial impact on mothers receiving expanded NBS results, as well as some of the research on NBS for lysosomal storage diseases mentioned earlier in this review.

Page 22, lines 376-379 – Not sure who you are including as stakeholders in this section, but you may find it interesting to compare with the results in Davids et al 2021 article asking about the use of NBS for reproductive choices for family members regarding Pompe disease (Health care practitioners’ experienced-based opinions on providing care after a positive NBS for Pompe)

Page 22, lines 390-393 – Please clarify the wording of these sentences to make clear that the percentages you report only pertain to the NBS-participating-parents, not the entire group of respondents.

Page 24, Conclusions section - Please clarify the wording of the first sentence to make clear that the conclusions you report only pertain to the NBS-participating-parents, not the entire group of respondents. Any other conclusion is confounded by the vast difference is sample size between groups.

**Supplementary Tables & Figures**

Table S1: Please include p-value or similar analytical statistic to allow reader to determine whether difference between columns is significant or not. You might also consider adding a column to show how many participants checked “I don’t know” as opposed to answering incorrectly, since both choices were given to participants.

Table S2: Please include p-value or similar analytical statistic to allow reader to determine whether difference between columns is significant or not.

Table S3: Please include p-value or similar analytical statistic to allow reader to determine whether difference between columns is significant or not.
